# Research hotspots and emerging trends in MicroRNA therapy for neuropathic pain: a bibliometric analysis (2009–2024)

**DOI:** 10.3389/fnmol.2025.1610844

**Published:** 2025-06-26

**Authors:** Yiqiang Zhang, Shushu Chen, Jiangtao Chi, Qinghua Gao, Yujie Li, Huaizhao Wang, Yuanyuan Yu

**Affiliations:** ^1^Discipline of Anesthesiology, Qingdao Medical College, Qingdao University, Qingdao, China; ^2^Department of Anesthesiology, Huangdao District Central Hospital, Qingdao, China; ^3^Department of Anesthesiology, Qingdao Hiser Hospital Affiliated of Qingdao University(Qingdao Traditional Chinese Medicine Hospital), Qingdao, China

**Keywords:** microRNA, neuropathic pain, therapy, bibliometrics, visualization analysis

## Abstract

**Objective:**

This study systematically investigates the evolving trends, research hotspots, and future directions in microRNA-based therapy for neuropathic pain (NP) through bibliometric analysis.

**Methods:**

We extracted literature related to microRNA interventions in NP from the Web of Science Core Collection database, spanning January 2009 to December 2024. A comprehensive analysis was conducted on publication trends, authorship patterns, institutional collaborations, national contributions, journal preferences, co-citation networks, and keyword clusters.

**Results:**

The final analysis included 250 articles, showing a steady increase in publications over the past 15 years. China was the most productive country, while the United States demonstrated the highest scientific influence. The top three institutions by publication count were Xuzhou Medical University, Nanjing Medical University, and the Chinese Academy of Medical Sciences and Peking Union Medical College. *Pain* was identified as the most co-cited journal. Keyword analysis revealed “differential expression” with the strongest citation burst intensity, while “peripheral nerve injury,” “mechanical allodynia,” and “proliferation” emerged as recent high-frequency terms.

**Conclusion:**

In the field of microRNA therapy for neuropathic pain, investigations into peripheral nerve injury mechanisms, neuroinflammation regulation, and miRNA differential expression patterns have been identified as current research hotspots. Emerging frontiers are now shifting toward three strategic directions: (1) development of targeted delivery systems, (2) precision modulation of nociceptive circuits, and (3) individualized therapeutic strategies. Collectively, miRNAs demonstrate significant potential as innovative NP treatments. While clinical translation of miRNA-based therapies remains a critical research priority, key challenges persist in optimizing target specificity (particularly sequence homology discrimination among miRNA isoforms) and ensuring biocompatibility of delivery platforms.

## Introduction

Neuropathic pain (NP), a chronic pain syndrome caused by damage or diseases of the peripheral or central nervous system, severely impairs patients’ quality of life. Its therapeutic management remains challenging, as first-line recommended medications often provide suboptimal relief for many patients. Conventional analgesics such as nonsteroidal anti-inflammatory drugs (NSAIDs) and opioids are limited by the development of tolerance with prolonged use. Current NP treatment strategies require a paradigm shift from merely suppressing symptoms to adopting disease-modifying approaches, including preventing adverse effects and reducing intrinsic risks. In recent years, microRNAs, critical regulators of gene expression, have emerged as a key research focus for NP therapy (Gada et al., 2022). MicroRNAs participate in the pathogenesis and progression of NP by modulating key biological processes, including neuronal excitability, neuroinflammatory cascades, and axonal regeneration (Jiang et al., 2022). Research indicates that miRNAs, abundantly expressed in the peripheral nervous system, modulate neuropathic pain pathomechanisms through targeted regulation of multiple signaling pathways and gene networks (Morchio et al., 2023). Consequently, miRNAs have emerged as promising therapeutic targets, offering novel directions for NP management through their ability to precisely regulate pathological networks.

## Literature and methods

### Literature sources and search methods

To ensure comprehensive and accurate data retrieval, we conducted an advanced search in the Web of Science Core Collection (SCI-EXPANDED/SSCI indices) using the formula TS = “neuropathic pain” AND TS = (“miRNA” OR “microRNA” OR “miRNAs” OR “MicroRNA” OR “RNA Micro”), with a time span from January 1, 2009, to December 31, 2024, restricted to English-language articles and reviews. This initial search served as inclusion criteria, followed by secondary screening that excluded publications lacking explicit references to “microRNA” and “neuropathic pain” in abstracts or those unrelated to miRNA therapeutic applications in NP. After removing duplicates, all bibliographic records—including titles, authors, abstracts, and citations—were systematically exported in plain text (TXT) format for subsequent analysis.

Bibliometrics, originating in the early 20th century, is an interdisciplinary field that applies mathematical and statistical techniques to quantify and analyze scholarly publications. Through bibliometric methods, researchers systematically extract metadata including authors, countries, journals, institutions, keywords, and cited references, while modern computational tools enable enhanced analytical depth through graphical visualization of complex academic networks.

Current research lacks comprehensive trend analyses on microRNA-based therapeutic development for NP. This study performs a bibliometric analysis of global scientific literature (2009–2024) to systematically elucidate research trajectories, emerging hotspots, and future innovation pathways in miRNA-mediated NP treatment strategies.

## Research methods

**Figure 1:**
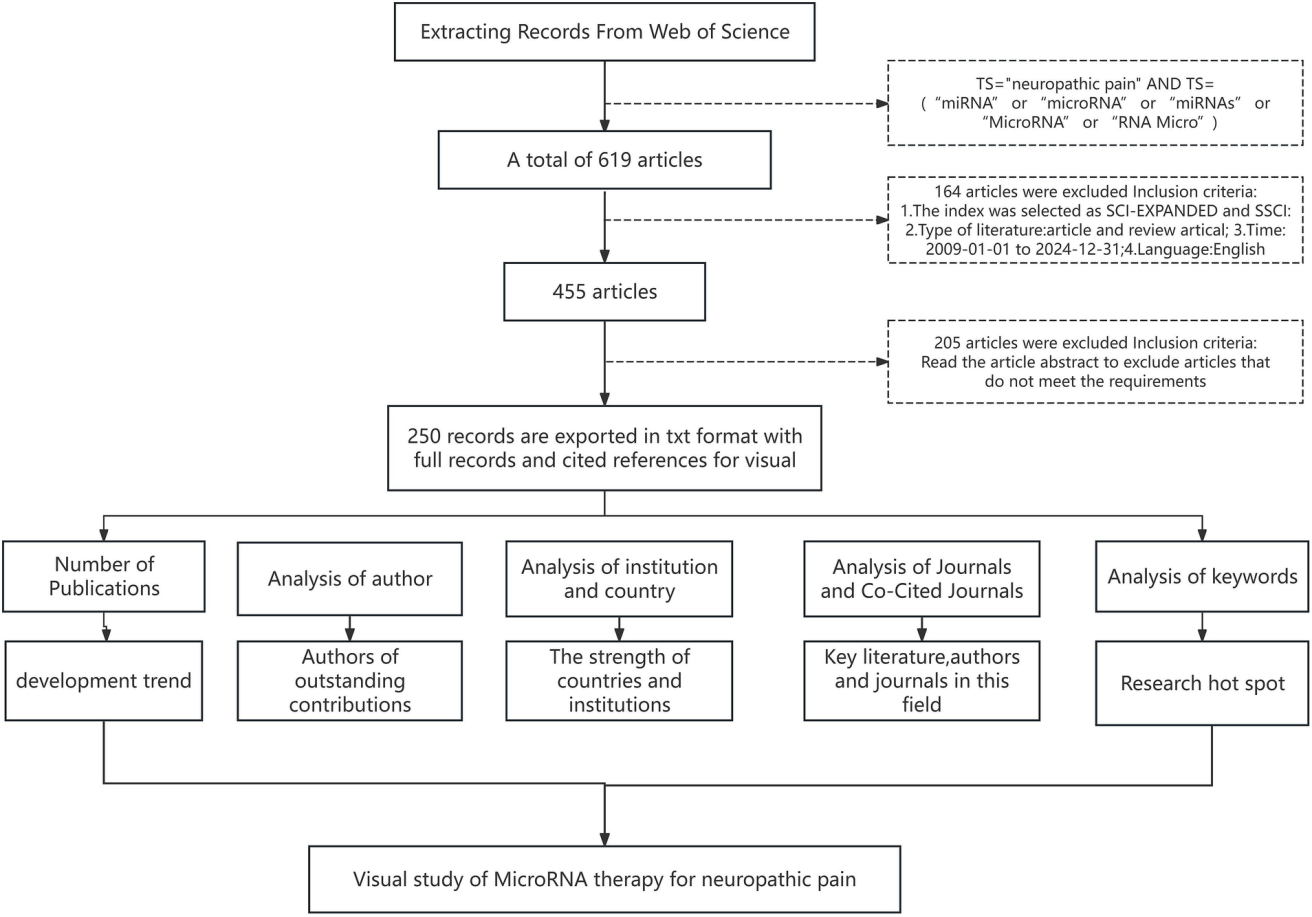
Flowchart of literature screening and analytical process.

CiteSpace, a scientific literature analysis tool co-developed by Dr. Chaomei Chen (Chinese-American scholar) and the WISE Laboratory, employs co-citation analysis and pathfinding network algorithms to visualize domain evolution patterns through data mapping. In CiteSpace, centrality analysis is grounded in network topology (e.g., collaboration/citation networks), with the core metric being Betweenness Centrality. This metric quantifies the criticality of a node as a “broker” or “mediator.” Its computational logic is as follows: Betweenness Centrality measures the frequency at which a node appears on the shortest paths between all pairs of nodes in the network. Specifically, it is calculated as the proportion of all shortest paths between node pairs that pass through the given node (typically normalized to the range [0, 1]). A higher value indicates a stronger “bridging” role of the node in connecting different subgroups, signifying greater hub prominence within the network. This platform generates scientific knowledge landscapes that delineate historical research trajectories, current hotspots, and emerging frontiers within specialized fields. Complementarily, VOSviewer—developed by Nees Jan van Eck and Ludo Waltman at Leiden University, Netherlands—specializes in constructing large-scale bibliometric networks using VOS mapping/clustering technologies. The software provides four visualization modes (label view, density view, cluster view, scatter view) with enhanced navigation features (zoom/scroll functionalities), enabling systematic exploration of complex academic relationships. In our study, synergistic application of CiteSpace and VOSviewer facilitated multidimensional analyses of authorship patterns, institutional collaborations, national contributions, journal impacts, keyword clusters, and co-cited references. The integrated methodological workflow is presented in Figure 1.

## Results

### Analysis of the number of publications

**Figure 2:**
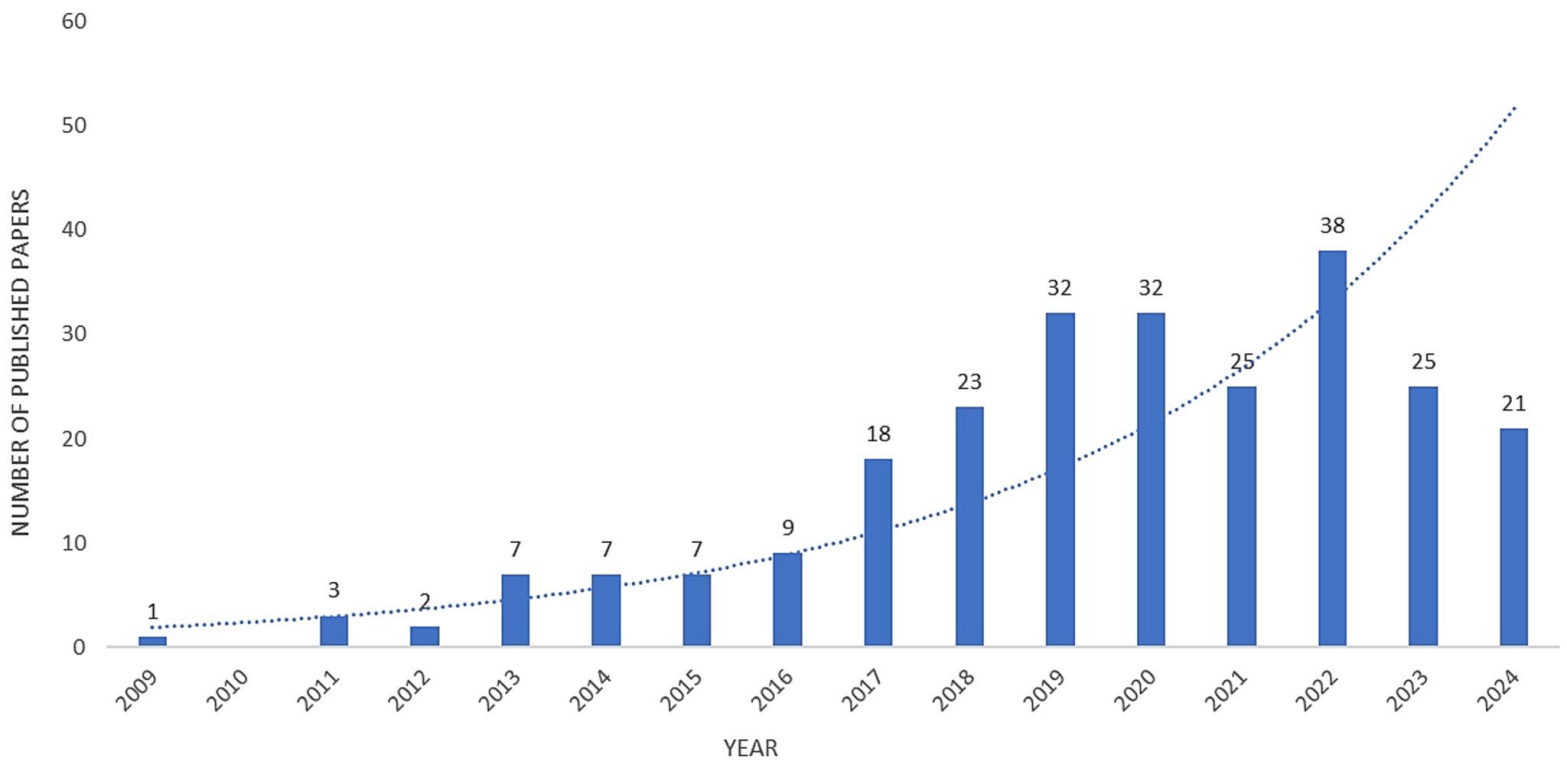
Annual publication volume on microRNA-based therapeutics for neuropathic pain.

As depicted in Figure 2, our search identified 250 publications, including 25 review articles. From 2009 to 2024, the annual output of microRNA-related publications in NP management demonstrated a sustained upward trajectory, peaking in 2022 (n = 37) followed by 2019 (n = 35). Notably, 138 articles (55% of total publications) were published during the recent quinquennium (2020–2024), reflecting accelerated research activity in miRNA-based NP therapeutics.

### Analysis of authors and co-cited authors

**Figure 3:**
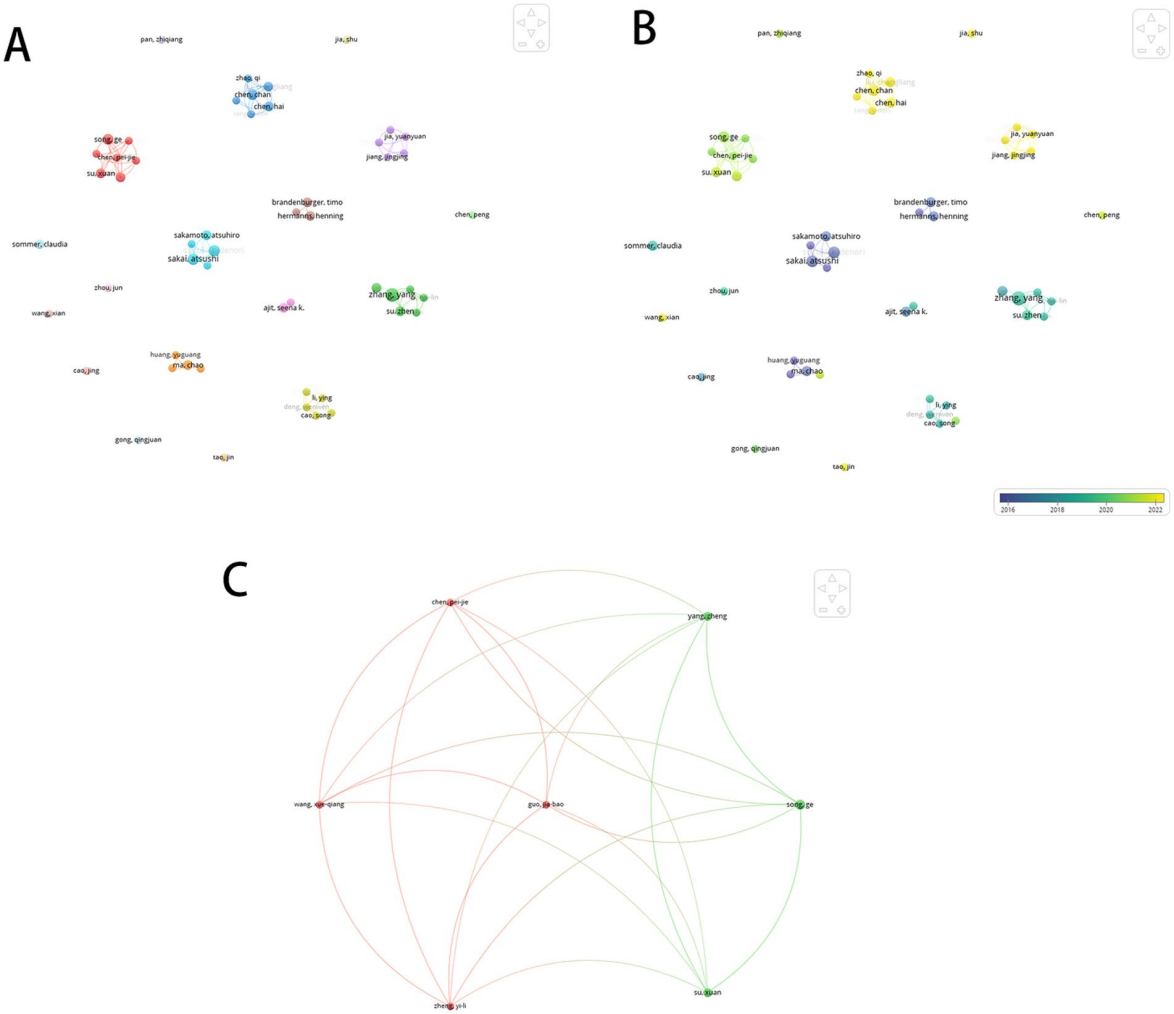
Author co-occurrence analysis. **(A)** Cluster visualization of author co-occurrence network. **(B)** Labeled collaboration network of authors. **(C)** Co-authorship network of top productive authors.

**Figure 4:**
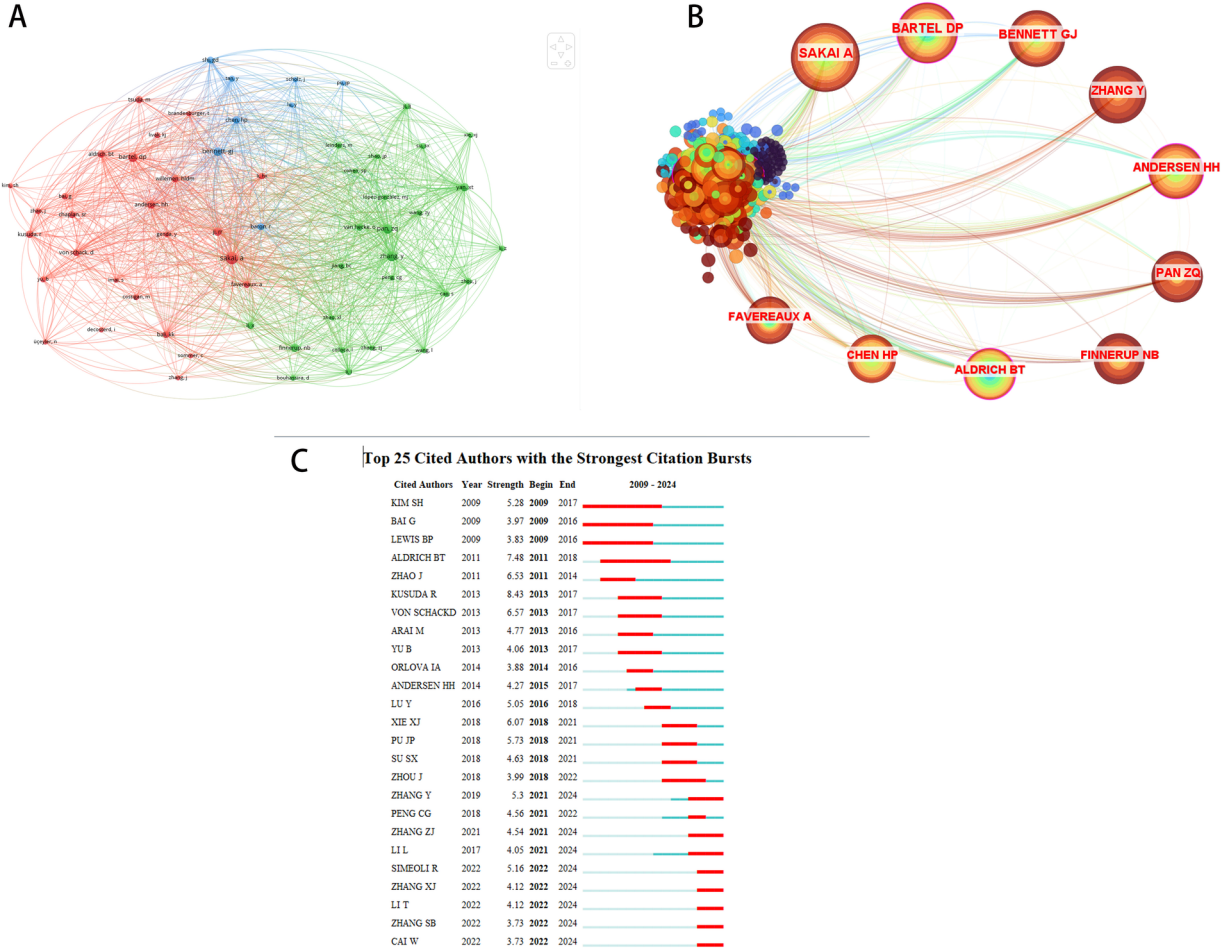
Co-citation network analysis of authors. **(A)** Cluster visualization of co-cited authors’ network. **(B)** Centrality-based co-occurrence network of co-cited authors. **(C)** Top 25 most bursting co-cited authors.

A total of 1,481 authors contributed to the 250 publications, with the top 10 most productive authors listed in Table 1. Atsushi Sakai, Yang Zhang, and Hidenori Suzuki each published six articles, receiving 355, 232, and 355 citations, respectively. Six of the top 10 authors were affiliated with Chinese institutions, followed by three from Japan and one from the United States. Further analysis of 52 authors with ≥3 publications revealed stable collaborative clusters through co-authorship visualization (Figure 3). Among 7,171 co-cited authors, 59 meeting the citation threshold (≥20 citations) were mapped in Figure 4. Table 2 presents the top 10 co-cited authors by citation frequency and centrality. Atsushi Sakai ranked highest in citations (76), while James Baig exhibited the greatest centrality (0.26), indicating their pivotal influence. Citation burst analysis identified three authors with the strongest burst intensities: Kusuda R (2013–2017), Aldrich BT (2011–2018), and von Schack D (2013–2017), whose sustained scholarly activity suggests continued contributions to this field.

**Table 1:** Top 10 contributing authors in microRNA-based therapeutics for neuropathic pain research.

Rank	Author	Counts	Citations	Country
1	Sakai Atsushi	6	355	Japan
2	Zhang Yang	6	232	China
3	Suzuki, Hidenori	6	355	Japan
4	Guo, Jiabao	5	74	China
5	Xueqiang Wang	5	74	China
6	Zhen,yi,li	5	74	China
7	Sommer Claudia	5	183	USA
8	Lu, Jingmin	4	227	China
9	Sakamoto, Atsuhiro	4	116	Japan
10	Chen, Hai	4	20	China

**Table 2:** Top 10 most frequently co-cited authors in microRNA-based therapeutics for neuropathic pain.

Rank	Co-cited author	Fre	Co-cited author	Centrality
1	Sakai A	76	Bai G	0.26
2	Bartel DP	59	Andersen HH	0.15
3	Bennett GJ	52	Baron R	0.13
4	Zhang Y	51	Bartel DP	0.11
5	Andersen HH	46	Sakai A	0.1
6	Pan ZQ	42	Aldrich BT	0.1
7	Finnerup NB	39	Alloui A	0.1
8	Aldrich BT	39	Abramoff M	0.1
9	Chen HP	37	Costigan M	0.08
10	Favereaux A	36	Bennett GJ	0.07

### Country and institution analysis

**Figure 5:**
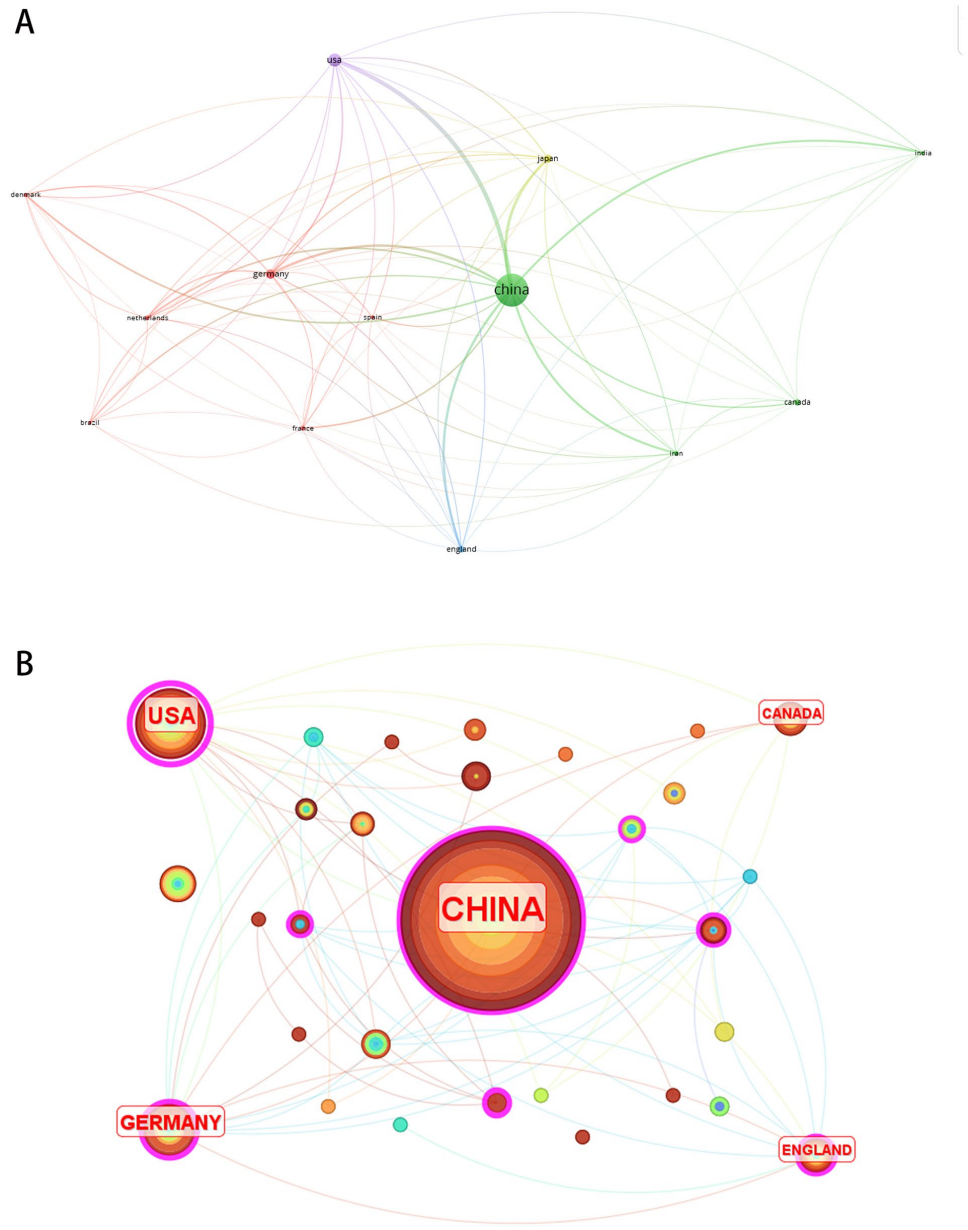
Country co-occurrence analysis. **(A)** Cluster visualization of country collaboration network. **(B)** Country-centric co-occurrence network.

Research teams from 31 countries contributed to the 250 publications. Figure 5 visualizes 13 nations with ≥3 articles. Table 3 lists the top 10 countries by publication volume and centrality. China dominated research output with 117 publications, demonstrating high productivity in miRNA-based NP therapeutics, though its low centrality (0.18) reflected limited international collaboration. In contrast, the United States ranked second in output (29 articles) yet achieved the highest centrality (0.59), indicating its pivotal role in global cooperative networks. These metrics reveal China’s quantitative leadership versus the U. S.’s qualitative influence in scholarly impact.

**Table 3:** Top 10 contributing countries in research on microRNA-based therapeutics for neuropathic pain.

Rank	Country	Counts	Country	Centrality
1	China	181	USA	0.63
2	USA	29	Germany	0.39
3	Germany	16	Croatia	0.24
4	Japan	11	China	0.19
5	Canada	7	France	0.17
6	England	7	England	0.14
7	France	5	Italy	0.14
8	Iran	5	Australia	0.1
9	Netherlands	5	Canada	0.04
10	Denmark	4	Denmark	0.04

**Figure 6:**
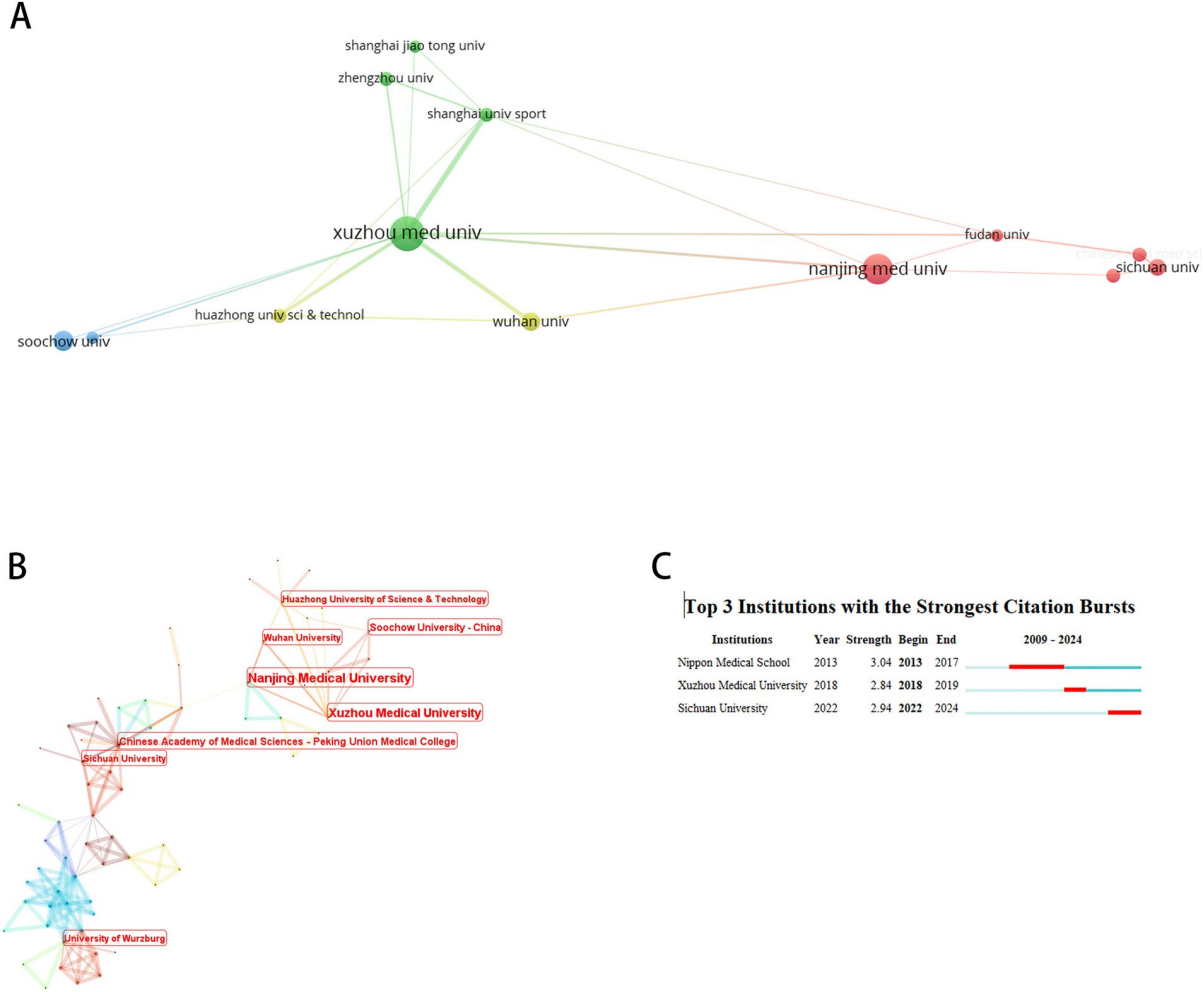
Institutional co-occurrence analysis. **(A)** Cluster Visualization of institutional collaboration network. **(B)** Centrality-based co-occurrence network of institutions. **(C)** Top 3 institutions with strongest citation bursts.

A total of 362 institutions participated in this field. Figure 6 maps institutions with ≥5 publications, while Table 4 details the top 10 by productivity and centrality. Xuzhou Medical University emerged as the most prolific institution. Notably, eight of the top 10 institutions were Chinese, with the Chinese Academy of Medical Sciences and Peking Union Medical College and Institut National de la Santé et de la Recherche Médicale (Inserm) exhibiting the highest centrality. Burst analysis identified Japanese medical schools as early contributors (peak burst: 2013–2017), whereas Sichuan University demonstrated recent dominance (surge period: 2022–2024).

**Table 4:** Top 10 research institutions in microRNA-based therapeutics for neuropathic pain.

Rank	Institutions	Counts	Country	Institutions	Centrality	Country
1	Xuzhou Medical University	14	China	Chinese Academy of Medical Sciences - Peking Union Medical College	0.11	China
2	Nanjing Medical University	13	China	Institut National de la Sante et de la Recherche Medicale (Inserm)	0.11	France
3	Chinese Academy of Medical Sciences - Peking Union Medical College	10	China	Nanjing Medical University	0.09	China
4	Soochow University - China	8	China	Fudan University	0.09	China
5	Wuhan University	7	China	Centre National de la Recherche Scientifique (CNRS)	0.09	France
6	Huazhong University of Science and Technology	7	China	Xuzhou Medical University	0.05	China
7	University of Wurzburg	7	Germany	University of Wurzburg	0.03	Germany
8	Sichuan University	7	China	Huazhong University of Science and Technology	0.02	China
9	Nippon Medical School	7	Japan	Wuhan University	0.01	China
10	Fudan University	6	China	Nanchang University	0.01	China

### Journal analysis

**Figure 7:**
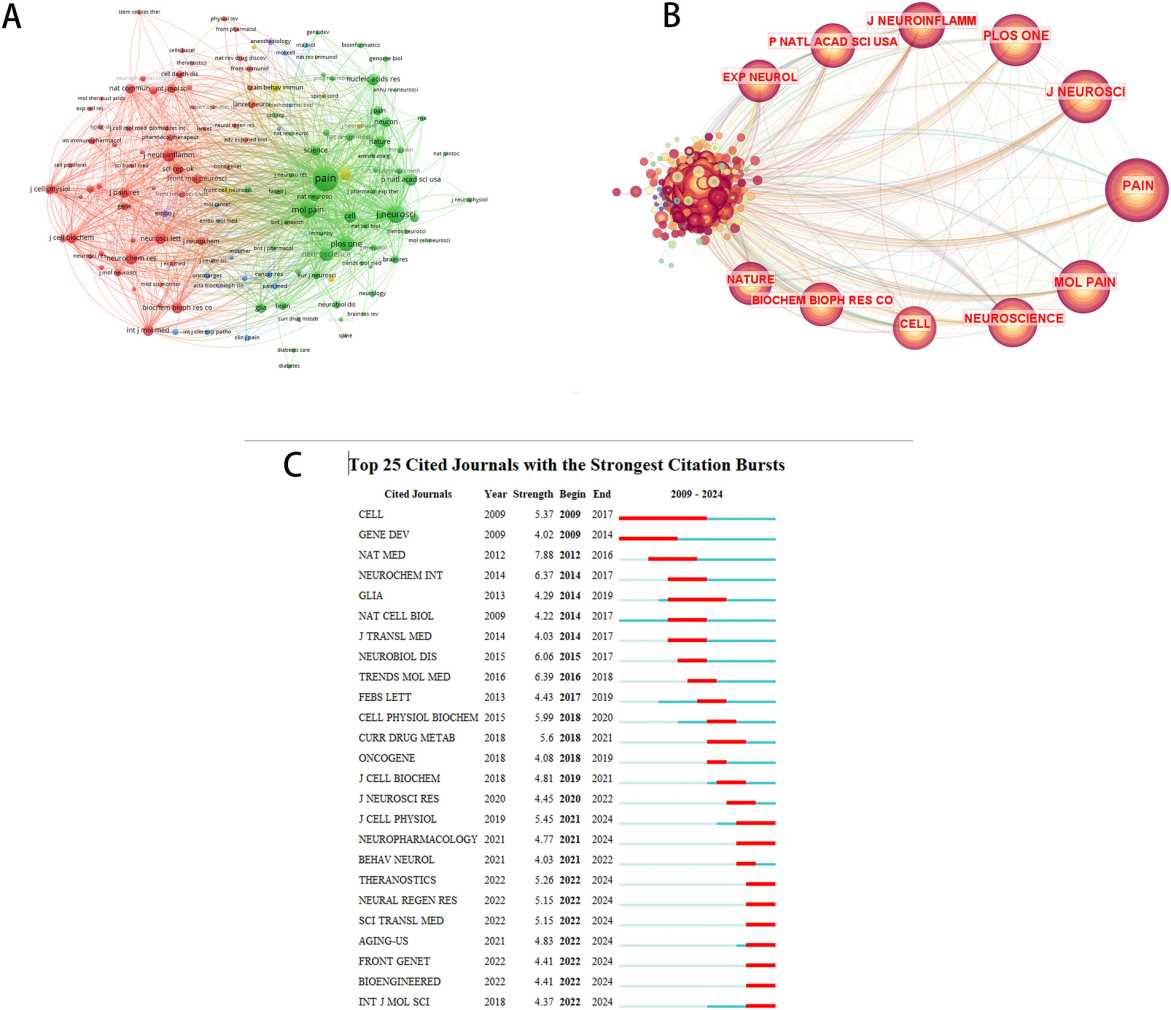
Co-citation network analysis of journals. **(A)** Cluster visualization of co-cited journals. **(B)** Centrality-based co-occurrence network of co-cited journals. **(C)** Top 25 co-cited journals with strongest citation bursts.

The top 10 journals by co-citation frequency and centrality are presented in Table 5. *The Journal of Pain* emerged as the most cited publication, accumulating 200 total citations, followed by *The Journal of Neuroscience (JNEUROSCI)* with 148 citations. *Proceedings of the National Academy of Sciences of the United States of America (PNAS)* exhibited the highest centrality score (0.08). Furthermore, *Nature Medicine (NAT MED)* demonstrated the strongest citation burst intensity (2012–2016), as visualized in Figure 7. These findings underscore the prominence of pain and neuroscience journals in shaping research priorities, highlighting their pivotal role in advancing therapeutic innovations for NP.

**Table 5:** Top 10 most cited journals in microRNA-based therapeutics for neuropathic pain.

Rank	Co-cited journal	Counts	IF(2023)	Co-cited journal	Centrality	IF(2023)
1	Pain	200	5.9	Proceedings of the National Academy of Sciences of the United States of America	0.08	9.4
2	Journal of Neuroscience	148	4.4	Journal of Neuroinflammation	0.06	9.3
3	Molecular Pain	146	2.8	Neuroscience Letters	0.06	2.5
4	Plos One	139	2.9	Glia	0.06	5.4
5	Neuroscience	121	2.9	Brain Research	0.06	2.7
6	Journal of Neuroinflammation	111	9.3	Journal of Neuroscience	0.05	4.4
7	Cell	106	45.6	Neurochemical Research	0.05	3.7
8	Experimental neurology	103	4.6	Scientific Reports	0.05	3.8
9	Proceedings of the National Academy of Sciences of the United States of America	94	9.4	Nature Neuroscience	0.05	21.3
10	Biochemical and Biophysical Research Communications	92	2.5	BRAIN	0.05	11.9

### Analysis of co-cited literature

Table 6 presents the top 10 co-cited articles in our analysis, with the study by Pan ZQ ranking highest in citation frequency (24 citations), while Kusuda R exhibited the strongest citation burst strength (9.15, 2013–2016). Sakai A contributed one article within this cohort. Among these publications, six focused on animal experiments and four were review articles. Notably, five of the six animal studies investigated targeted signaling pathways, with the remaining study comparing differential expression of a specific microRNA between human and rat NP groups versus controls. All analyzed works addressed miRNA-based NP therapeutics, emphasizing the roles of distinct miRNAs in modulating peripheral nervous system pathophysiology and nociceptive circuit regulation.

**Table 6:** Top 10 most cited articles.

Rank	Author	Counts	Centrality
1	Pan ZQ, (2018)	24	0.06
2	Andersen HH, (2014)	23	0.05
3	Chen HP, (2014)	22	0.13
4	Su SX, (2017)	21	0.02
5	Peng CG, (2017)	19	0.08
6	López-González MJ, (2017)	19	0
7	Wang ZY, (2018)	18	0.01
8	Kusuda R, (2011)	18	0.07
9	Leinders M, (2016)	18	0.03
10	Sakai A, (2014)	18	0.02

**Figure 8:**
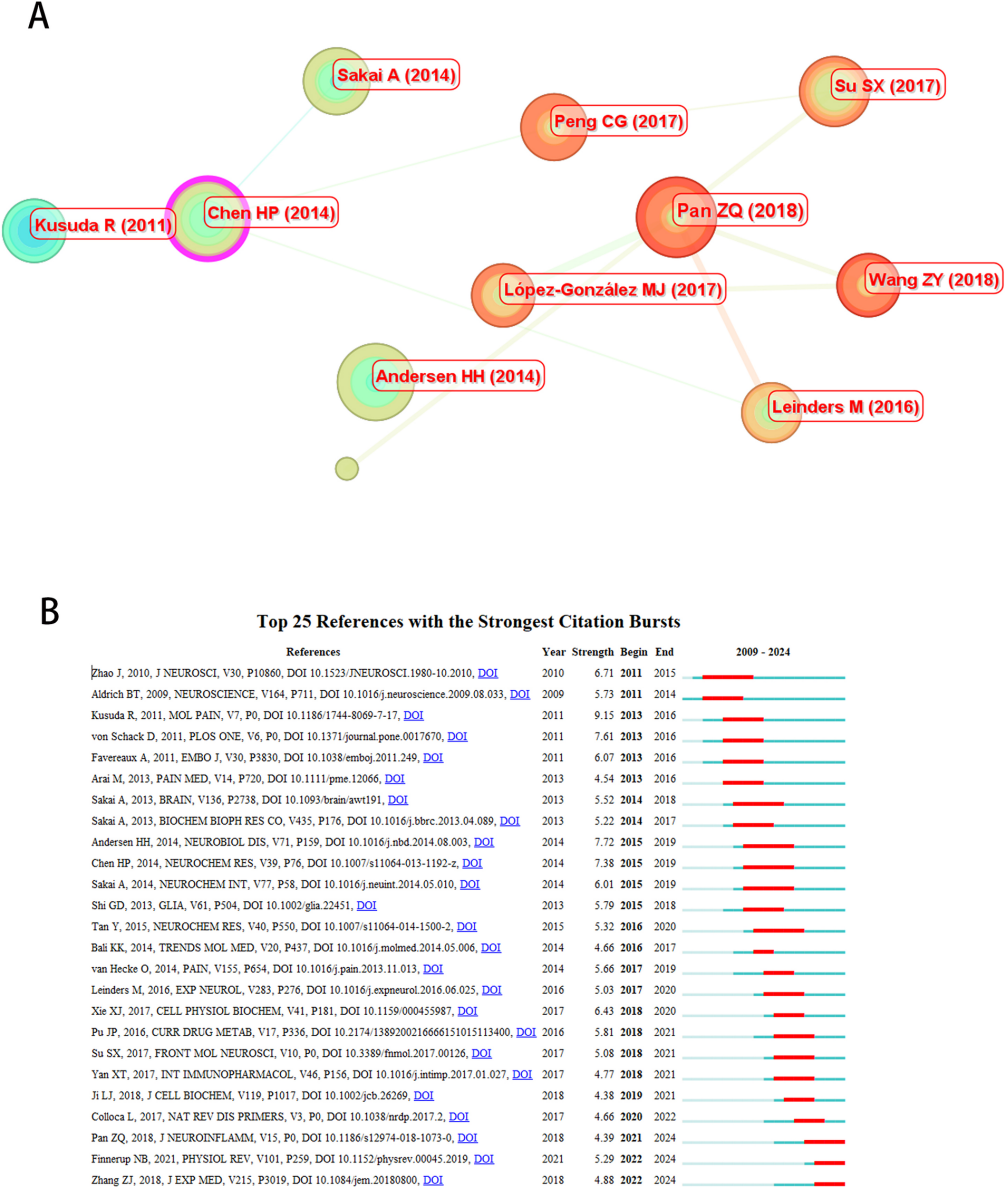
Co-citation analysis of cited articles. **(A)** Co-citation network of top 10 cited articles. **(B)** Top 25 references with strongest citation bursts.

Figure 8A illustrates the co-citation network of the 10 most-cited articles, where node size corresponds to citation frequency, and color gradation reflects temporal proximity (warmer hues indicating recency). Four articles published pre-2017 primarily explored foundational miRNA discoveries, including expression profiling and biomarker potential. In contrast, six post-2017 publications emphasized advanced mechanistic investigations (e.g., multi-target pathway dissection) and therapeutic innovations (e.g., targeted delivery systems), mirroring the field’s technological maturation. Figure 8B lists the 25 articles with the highest citation burst intensities, further delineating temporal shifts in research priorities.

### Keyword analysis

**Figure 9:**
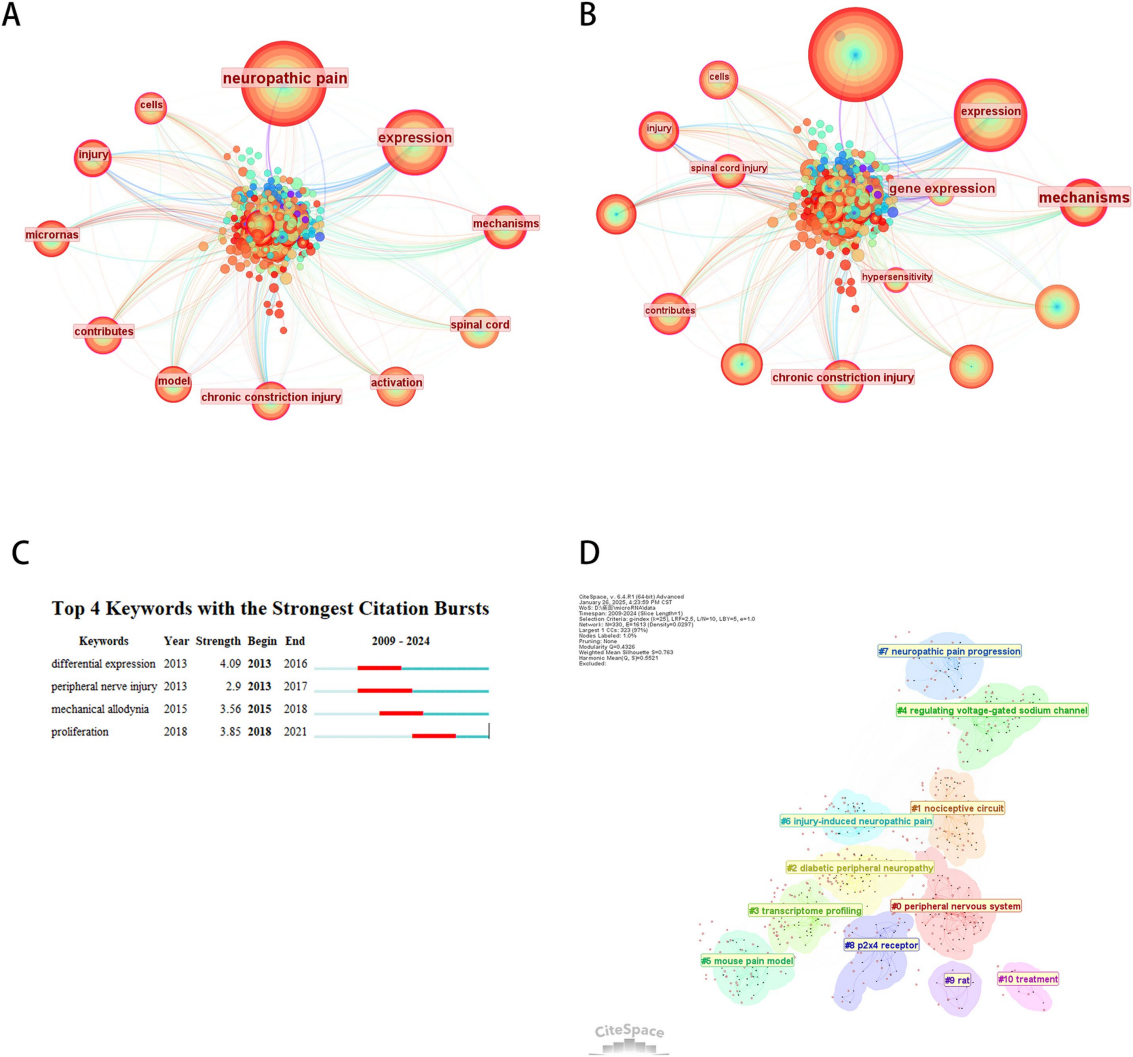
Keyword co-occurrence analysis. **(A)** Keyword co-occurrence network. **(B)** Centrality-based co-occurrence network of keywords. **(C)** Top 4 keywords with strongest term bursts. **(D)** Keyword clustering network.

As shown in Figure 9, 273 keywords were identified through co-occurrence analysis, with the top 10 keywords by frequency and centrality listed in Table 7. Excluding “neuropathic pain,” the most frequent and central terms were “expression,” “mechanism,” and “spinal cord.” These findings demonstrate that *differential expression* and *peripheral nerve injury* represent dominant research hotspots, with most studies focusing on these themes. Burst detection analysis identified four keywords with notable citation bursts, among which *differential expression* exhibited the highest burst strength (4.17). Temporal burst tracking revealed intensified interest in recent years for *differential expression* (peak burst: 4.91), *peripheral nerve injury* (3.26), and *mechanical allodynia* (4.05). We performed keyword clustering based on their co-occurrence proximity, resulting in 10 cluster labels. Smaller cluster numbers indicate larger keyword groupings, with the most extensive cluster labeled *peripheral nervous system (#0)*, followed by *nociceptive circuits (#1)* and *diabetic peripheral neuropathy (#2)*.

**Table 7:** Top 10 keywords in microRNA-based therapeutics for neuropathic pain.

Rank	Keywords	Counts	Keywords	Centrality
1	Neuropathic pain	164	Mechanisms	0.18
2	Expression	99	Expression	0.17
3	Mechanisms	41	Gene expression	0.14
4	Spinal cord	38	Contributes	0.13
5	Activation	37	Cells	0.12
6	Chronic constriction injury	37	Spinal cord injury	0.12
7	Model	33	Dorsal root ganglion	0.12
8	Contributes	33	Model	0.11
9	Micrornas	32	Injury	0.11
10	Injury	31	Hypersensitivity	0.11

## Discussion

This bibliometric analysis of microRNA-based therapeutics for NP revealed sustained growth in publication output over the past 15 years, accompanied by expanding research engagement in this domain. *Pain*, *Neuroscience*, and *Molecular Pain* em0erged as the three most frequently cited journals, indicating concentrated scholarly exploration of miRNA-mediated NP interventions within neuroscience and molecular pain research frameworks.

Analysis of annual publication trends revealed a marked increase in research output commencing in 2017, enabling chronological stratification of the field into pre-2017 and post-2017 phases characterized by differential growth patterns.

Prior to 2017, research predominantly focused on the regulatory roles and molecular mechanisms of microRNAs in neuropathic pain (NP). Studies extensively investigated how various miRNAs modulated pain perception and transmission through targeted genes or signaling pathways. Atsushi Sakai and Hidenori Suzuki emerged as the most prolific contributors during this period, with their collaborative network demonstrating concentrated productivity before 2017. Their seminal work revealed critical miRNA involvement in NP pathogenesis, exemplified by a 2013 study showing that miR-7a significantly alleviated neuropathic pain maintenance via neuronal excitability regulation. Notably, miR-7a exhibited a pain-selective effect, specifically mitigating NP without influencing physiological or inflammatory pain states (Sakai et al., 2013). In their subsequent 2017 study, they demonstrated that the miR-17-92 cluster reduces potassium currents through synergistic modulation of multiple voltage-gated potassium channels and associated regulatory subunits (Sakai et al., 2017), Specifically, A-type currents contribute to the maintenance of neuropathic pain. In the same year, their collaborative study further demonstrated that miR-15b participates in oxaliplatin-induced chronic neuropathic pain through downregulating BACE1 expression (Ito et al., 2017; Yang et al., 2023). These studies provide critical evidence supporting the specific role of miRNA in neuropathic pain (Sakai and Suzuki, 2015).

Furthermore, the research also investigated the alterations in miRNA expression profiles following nerve injury (Gong et al., 2015; Ikuma et al., 2023; Kowalski et al., 2022), Specifically focusing on their expression in the dorsal root ganglia and spinal cord, as well as the dynamic alterations of miRNAs observed in chronic pain models such as diabetic neuropathic pain and sciatic nerve compression injury (Wilkerson et al., 2020; Zheng et al., 2022). Through establishing distinct pain models (including chronic inflammatory pain, neuropathic pain, and acute nociceptive stimulation paradigms), Kusuda R revealed differential miRNA expression patterns in the dorsal root ganglion (DRG) and spinal dorsal horn. The study demonstrated that miRNA expression exhibits not only temporal and spatial specificity but also stimulus-dependent regulation. These findings provide crucial insights into molecular mechanisms underlying various pain states and lay a foundation for developing miRNA-based diagnostic approaches and therapeutic strategies for pain management.

Since 2017, research focus has progressively shifted to exploring the role of extracellular vesicles (EVs) in neuropathic pain, particularly their miRNA-mediated regulation of microglial and astrocytic activation. Kexin Zhang’s research has further elucidated the therapeutic potential of EVs in neuropathic pain management. EVs demonstrate remarkable capacity to transport diverse molecular cargo (including proteins, DNA, mRNAs, and non-coding RNAs), serving as effective vectors for non-coding RNA delivery. This discovery unveils novel therapeutic possibilities for neuropathic pain treatment. The study also proposes innovative therapeutic strategies such as nanoparticle-mediated miRNA delivery and stem cell-derived exosome therapies, establishing new directions for developing RNA-based precision therapeutics (Bhandari et al., 2022; Gao et al., 2023; Lu et al., 2024; Luo et al., 2023; Poongodi et al., 2024; Zhang et al., 2023; Zhang et al., 2021). The research team led by Lu Yitian has developed a nanoparticle-based miRNA delivery system that effectively addresses the challenge of short *in vivo* half-life of miRNAs by encapsulating therapeutic miRNAs within functionalized nanoparticles with targeting capabilities. This innovative approach significantly enhances targeted delivery efficiency to specific cells. Utilizing microglia-specific targeting, the nanoparticle system loaded with miR-26a-5p achieves sustained analgesic effects in chronic pain management, demonstrating substantial clinical potential for long-term pain relief strategies.

Looking ahead, while miRNA-based personalized therapy shows immense potential, it still faces substantial challenges such as poor in vivo stability of miRNAs, inadequate targeting specificity and delivery efficiency of current systems, and difficulties in translating laboratory findings into clinical applications. Recent literature indicates that miR-155 has established a complete chain of evidence, spanning from mechanistic validation to therapeutic potential, across diverse models including Diabetic Peripheral Neuropathy (DPN), bortezomib/oxaliplatin (BTZ/OXL) chemotherapy-induced neurotoxicity, Spinal Cord Injury (SCI), Chronic Constriction Injury (CCI), and Regulatory T cell (Tregs) differentiation. Concurrently, miR-146a-5p has been mechanistically validated in CCI/Spinal Nerve Ligation (SNL) models and demonstrated long-lasting analgesia persisting for days following nanoparticle delivery in the Spared Nerve Injury (SNI) model, underscoring the translational value of sustained-release drug delivery strategies. Notably, significant mechanistic controversies exist within the field. For instance, miR-101 exerts analgesic effects by inhibiting mTOR signaling in the CCI model, whereas in another study, it exacerbates hyperalgesia via the MKP-1 pathway. Similarly, miR-21-5p exhibits pro-nociceptive effects in the SNI model yet demonstrates analgesic effects in the CCI model. These paradoxical findings suggest that model-specific differences and tissue-specific expression patterns may be key contributing factors. Golmakani’s research has delineated the roles of newly identified miRNAs in neuropathic pain, demonstrating that upregulated or downregulated miRNA expression patterns can modulate pain progression by controlling neural regeneration, neuroinflammation, and abnormal ion channel expression. With deeper mechanistic understanding of miRNAs and the application of advanced technologies like miRNA microarrays and high-throughput methodologies, miRNAs may emerge as novel biomarkers for disease diagnosis while offering promising therapeutic targets and strategies for neuropathic pain management (Andersen et al., 2014; Golmakani et al., 2024). Research by Michaela Kress has demonstrated that miRNAs play pivotal roles in nociceptive circuits by regulating signal transmission among neurons, glial cells, and immune cells. miRNAs may act as “master switches” in modulating chronic pain, particularly in the pathophysiology of neuropathic pain, migraine, and complex regional pain syndrome (CRPS) (Kress et al., 2013).

Current research still faces significant limitations: clinical data are scarce (only a limited number of studies report relevant findings), animal experiments predominantly rely on the intrathecal injection route, and the understanding of miRNA regulatory networks remains incomplete. Future studies should focus on insufficiently validated miRNAs, leveraging nanotechnology and multi-omics analyses to establish spatiotemporally-precise regulatory systems. More importantly, establishing collaborative networks across laboratories to integrate clinical samples from neuropathic pain patients with diverse etiologies is essential. This will enable the construction of standardized miRNA expression profile databases. Shared access to these data resources will accelerate the resolution of mechanistic controversies and the optimization of clinical translation pathways.

In summary, significant advancements have been achieved in miRNA research within the field of neuropathic pain. Future investigations will increasingly emphasize precision and personalization approaches while accelerating the translation of laboratory findings into clinical applications (Zhang et al., 2022; Zhou et al., 2017). The exploration of miRNAs as diagnostic biomarkers, clinical translation of targeted therapies, multi-target combination therapies, regulation of neuroinflammation and neuroregeneration, and precise modulation of nociceptive circuits will emerge as key research priorities. These investigations are expected to yield novel approaches and strategies for neuropathic pain diagnosis and treatment, ultimately advancing the clinical translation of miRNA-based interventions from laboratory research to practical therapeutic applications.

## Conclusion

This study conducted a comprehensive bibliometric analysis of microRNAs in NP, including in-depth evaluations of publications, authors, institutions, countries, journals, references, and keywords. The results demonstrate that miRNAs’ role in neuropathic pain has garnered increasing scholarly attention, with a stable collaborative network established among international researchers. The United States emerged as the most influential nation in this research domain, while *Pain* was identified as the most frequently cited journal. Current research hotspots focus on mechanisms of peripheral nerve injury and differential miRNA expression patterns. Future directions will emphasize the role of dorsal root ganglia in pain signal transmission, personalized therapeutic approaches, and targeted delivery systems. Specifically, the precise modulation of nociceptive circuits for treating chronic neuropathic pain is projected to dominate research priorities. However, these research avenues face persistent challenges, including miRNA target specificity and multi-target effects, biocompatibility and tissue penetration capacity of delivery systems, and feasibility of clinical translation.

## Data Availability

The raw data supporting the conclusions of this article will be made available by the authors, without undue reservation.

## References

[ref1] Andersen H. H. Duroux M. Gazerani P. (2014). Micrornas as modulators and biomarkers of inflammatory and neuropathic pain conditions. Neurobiol. Dis. 71, 159–168. doi: 10.1016/j.nbd.2014.08.003, PMID: 25119878

[ref2] Bhandari R. Sharma A. Kuhad A. (2022). Novel Nanotechnological approaches for targeting dorsal root ganglion (Drg) in mitigating diabetic neuropathic pain (Dnp). Front. Endocrinol. 12:790747. doi: 10.3389/fendo.2021.790747, PMID: 35211091 PMC8862660

[ref3] Gada Y. Pandey A. Jadhav N. Ajgaonkar S. Mehta D. Nair S. (2022). New vistas in microrna regulatory Interactome in neuropathic pain. Front. Pharmacol. 12:778014. doi: 10.3389/fphar.2021.778014, PMID: 35280258 PMC8914318

[ref4] Gao X. Gao L.-F. Kong X.-Q. Zhang Y. N. Jia S. Meng C. Y. (2023). Mesenchymal stem cell-derived extracellular vesicles carrying miR-99b-3p restrain microglial activation and neuropathic pain by stimulating autophagy. Int. Immunopharmacol. 115:109695. doi: 10.1016/j.intimp.2023.109695, PMID: 36638658

[ref5] Golmakani H. Azimian A. Golmakani E. (2024). Newly discovered functions of mirnas in neuropathic pain: transitioning from recent discoveries to innovative underlying mechanisms. Mol. Pain 20:20. doi: 10.1177/17448069231225845, PMID: 38148597 PMC10851769

[ref6] Gong Q. Lu Z. Huang Q. Ruan L. Chen J. Liang Y.. (2015). Altered micrornas expression profiling in mice with diabetic neuropathic pain. Biochem. Biophys. Res. Commun. 456, 615–620. doi: 10.1016/j.bbrc.2014.12.004, PMID: 25498543

[ref7] Ikuma Y. Sakai A. Sakamoto A. Suzuki H. (2023). Increased extracellular release of micrornas from dorsal root ganglion cells in a rat model of neuropathic pain caused by peripheral nerve injury. PLoS One 18:e0280425. doi: 10.1371/journal.pone.0280425, PMID: 36662897 PMC9858844

[ref8] Ito N. Sakai A. Miyake N. Maruyama M. Iwasaki H. Miyake K.. (2017). miR-15b mediates oxaliplatin-induced chronic neuropathic pain through Bace1 down regulation. Br. J. Pharmacol. 174, 386–395. doi: 10.1111/bph.13698, PMID: 28012171 PMC5301044

[ref9] Jiang M. Wang Y. Wang J. Feng S. Wang X. (2022). The etiological roles of mirnas, lncrnas, and circrnas in neuropathic pain: a narrative review. J. Clin. Lab. Anal. 36:e24592. doi: 10.1002/jcla.24592, PMID: 35808924 PMC9396192

[ref10] Kowalski J. L. Nguyen N. Battaglino R. A. Falci S. P. Charlifue S. Morse L. R. (2022). miR-338-5p levels and cigarette smoking are associated with neuropathic pain severity in individuals with spinal cord injury: preliminary findings from a genome-wide microrna expression profiling screen. Arch. Phys. Med. Rehabil. 103, 738–746. doi: 10.1016/j.apmr.2021.09.005, PMID: 34717922

[ref11] Kress M. Huettenhofer A. Landry M. Kuner R. Favereaux A. Greenberg D.. (2013). Micrornas in nociceptive circuits as predictors of future clinical applications. Front. Mol. Neurosci. 6:33. doi: 10.3389/fnmol.2013.00033, PMID: 24151455 PMC3798051

[ref12] Lu Y. Liu S. Wang P. Guo X. Qin Z. Hou H.. (2024). A novel microglia-targeting strategy based on nanoparticle-mediated delivery of miR-26a-5p for long-lasting analgesia in chronic pain. J. Nanobiotechnol. 22:128. doi: 10.1186/s12951-024-02420-9, PMID: 38519978 PMC10960380

[ref13] Luo X. Jean-Toussaint R. Tian Y. Balashov S. V. Sacan A. Ajit S. K. (2023). Small extracellular vesicles from spared nerve injury model and sham control mice differentially regulate gene expression in primary microglia. J. Pain 24, 1570–1581. doi: 10.1016/j.jpain.2023.03.015, PMID: 37044293 PMC10524046

[ref14] Morchio M. Sher E. Collier D. A. Lambert D. W. Boissonade F. M. (2023). The role of mirnas in neuropathic pain. Biomedicines 11:775. doi: 10.3390/biomedicines11030775, PMID: 36979754 PMC10045079

[ref15] Poongodi R. Yang T.-H. Huang Y.-H. Yang K. D. Chen H. Z. Chu T. Y.. (2024). Stem cell exosome-loaded Gelfoam improves locomotor dysfunction and neuropathic pain in a rat model of spinal cord injury. Stem Cell Res Ther 15:143. doi: 10.1186/s13287-024-03758-5, PMID: 38764049 PMC11103960

[ref16] Sakai A. Saitow F. Maruyama M. Miyake N. Miyake K. Shimada T.. (2017). Microrna cluster miR-17-92 regulates multiple functionally related voltage-gated potassium channels in chronic neuropathic pain. Nat. Commun. 8:16079. doi: 10.1038/ncomms16079, PMID: 28677679 PMC5504285

[ref17] Sakai A. Saitow F. Miyake N. Miyake K. Shimada T. Suzuki H. (2013). miR-7a alleviates the maintenance of neuropathic pain through regulation of neuronal excitability. Brain 136, 2738–2750. doi: 10.1093/brain/awt191, PMID: 23861446

[ref18] Sakai A Suzuki H. (2015). Microrna and pain Santulli G. Microrna: Medical Evidence: From Molecular Biology to Clinical Practice. 17–39.

[ref19] Wilkerson J. L. Jiang J. Felix J. S. Bray J. K. da Silva L. Gharaibeh R. Z.. (2020). Alterations in mouse spinal cord and sciatic nerve micrornas after the chronic constriction injury (cci) model of neuropathic pain. Neurosci. Lett. 731:135029. doi: 10.1016/j.neulet.2020.135029, PMID: 32380144 PMC7339614

[ref20] Yang X. Huang X. Lu W. Yan F. Ye Y. Wang L.. (2023). Transcriptome profiling of mirna-mrna interactions and associated mechanisms in chemotherapy-induced neuropathic pain. Mol. Neurobiol. 60, 5672–5690. doi: 10.1007/s12035-023-03398-5, PMID: 37332017

[ref21] Zhang K. Li P. Jia Y. Liu M. Jiang J. (2023). Concise review: current understanding of extracellular vesicles to treat neuropathic pain. Front. Aging Neurosci. 15:1131536. doi: 10.3389/fnagi.2023.1131536, PMID: 36936505 PMC10020214

[ref22] Zhang H.-G. Liu L. Song Z.-P. Zhang D. Y. (2022). Bioinformatics analysis of the Microrna-metabolic gene regulatory network in neuropathic pain and prediction of corresponding potential therapeutics. J. Mol. Neurosci. 72, 468–481. doi: 10.1007/s12031-021-01911-w, PMID: 34580818 PMC8476070

[ref23] Zhang Y. Liu J. Wang X. Zhang J. Xie C. (2021). Extracellular vesicle-encapsulated microrna-23a from dorsal root ganglia neurons binds to A20 and promotes inflammatory macrophage polarization following peripheral nerve injury. Aging 13, 6752–6764. doi: 10.18632/aging.202532, PMID: 33621204 PMC7993670

[ref24] Zheng Y.-L. Su X. Chen Y.-M. Guo J. B. Song G. Yang Z.. (2022). Microrna-based network and pathway analysis for neuropathic pain in rodent models. Front. Mol. Biosci. 8:780730. doi: 10.3389/fmolb.2021.780730, PMID: 35096965 PMC8794747

[ref25] Zhou J. Xiong Q. Chen H. Yang C. Fan Y. (2017). Identification of the spinal expression profile of non-coding Rnas involved in neuropathic pain following spared nerve injury by sequence analysis. Front. Mol. Neurosci. 10:91. doi: 10.3389/fnmol.2017.00091, PMID: 28420964 PMC5377590

